# Selected Plant Metabolites Involved in Oxidation-Reduction Processes during Bud Dormancy and Ontogenetic Development in Sweet Cherry Buds (*Prunus avium* L.)

**DOI:** 10.3390/molecules23051197

**Published:** 2018-05-17

**Authors:** Susanne Baldermann, Thomas Homann, Susanne Neugart, Frank-M. Chmielewski, Klaus-Peter Götz, Kristin Gödeke, Gerd Huschek, Getrud E. Morlock, Harshadrai M. Rawel

**Affiliations:** 1 Institute of Nutritional Science, University of Potsdam, Arthur-Scheunert-Allee 114-116, 14558 Nuthetal, Potsdam, Germany; Baldermann@igzev.de (S.B.); homann@uni-potsdam.de (T.H.); 2 Leibniz Institute of Vegetable and Ornamental Crops (IGZ), Theodor-Echtermeyer-Weg 1, 14979 Großbeeren, Germany; neugart@igzev.de; 3 Agricultural Climatology, Faculty of Life Sciences, Humboldt-University of Berlin, Albrecht-Thaer-Weg 5, 14195 Berlin, Germany; chmielew@agrar.hu-berlin.de (F.-M.C.); klaus-peter.goetz@agrar.hu-berlin.de (K.-P.G.); 4 IGV-Institut für Getreideverarbeitung GmbH, Arthur-Scheunert-Allee 40/41, 14558, Nuthetal OT Bergholz-Rehbrücke, Germany; kristin.goedeke@igv-gmbh.de (K.G.); gerd.huschek@igv-gmbh.de (G.H.); 5 Chair of Food Sciences, Institute of Nutritional Science, Interdisciplinary Research Center (IFZ), Justus Liebig University Giessen, Heinrich Buff Ring 26-32, D-35392 Giessen, Germany; Gertrud.Morlock@ernaehrung.uni-giessen.de

**Keywords:** dormancy, redox-metabolites, phenolics, ascorbate, anti-oxidative capacity, *Prunus avium* L., flower buds

## Abstract

Many biochemical processes are involved in regulating the consecutive transition of different phases of dormancy in sweet cherry buds. An evaluation based on a metabolic approach has, as yet, only been partly addressed. The aim of this work, therefore, was to determine which plant metabolites could serve as biomarkers for the different transitions in sweet cherry buds. The focus here was on those metabolites involved in oxidation-reduction processes during bud dormancy, as determined by targeted and untargeted mass spectrometry-based methods. The metabolites addressed included phenolic compounds, ascorbate/dehydroascorbate, reducing sugars, carotenoids and chlorophylls. The results demonstrate that the content of phenolic compounds decrease until the end of endodormancy. After a long period of constancy until the end of ecodormancy, a final phase of further decrease followed up to the phenophase open cluster. The main phenolic compounds were caffeoylquinic acids, coumaroylquinic acids and catechins, as well as quercetin and kaempferol derivatives. The data also support the protective role of ascorbate and glutathione in the para- and endodormancy phases. Consistent trends in the content of reducing sugars can be elucidated for the different phenophases of dormancy, too. The untargeted approach with principle component analysis (PCA) clearly differentiates the different timings of dormancy giving further valuable information.

## 1. Introduction

The monitoring of bud dormancy in temperate latitudes is becoming a necessary tool to improve fruit flowering for sweet cherry and the correct implementation of agricultural actions such as protection against frost damage. Three characteristic phases of dormancy (para-, endo-, and ecodormancy) are discussed. Many studies have been directed to determine the course of these phenophases [1,2,3,4,5], through which a central role for the phytohormone abscisic acid (ABA) has been proposed [1,3,6]. The leaf fall (LF) generally marks the end of paradormancy and the onset of endodormancy [2]. Subsequently, no visual changes can be noted on the tree to determine the transition from endo- to ecodormancy. The release from endodormancy requires a sufficiently long period of low temperatures providing the necessary chilling requirement and can be described by chilling models [2,4,7]. Twigs from naturally growing trees are regularly taken to observe their development under controlled environmental conditions, allowing one to determine the transition from endo- to ecodormancy (t_1_) by bud break [2,8]. It is a shortcoming of phenological models that the beginning of ontogenetic development cannot be accurately predicted. Generally, the end of ecodormancy, and consequently the beginning of ontogenetic development (t_1_*), is described by forcing models (2-phase models) [2,8].

Many biochemical processes are involved in regulating the consecutive transition of these different phases as determined by protein expression and identification [4]. The expression of many dehydrins, a family of ubiquitous proteins belonging to angiosperms and gymnosperms, can be induced by the phytohormone ABA [9]. Dehydrins help proteins to fold properly and/or prevent their aggregation under heat or freezing stress, providing cryoprotective activity. During investigations on seasonal changes in protein profiles in dormant flower buds of Japanese apricot (*Prunus mume Siebold and Zucc.*) cultivars, one protein, isolated by two-dimensional polyacrylamide gel electrophoresis of flower bud extracts, was shown by peptide sequencing to be a dehydrin [10]. Proteomic methods applied to follow the events from dormancy to release that occur during the apical bud development of *Pinus sylvestris* L. showed that the majority of these proteins (57%) are involved in metabolic and other cellular processes [11]. With regard to endodormancy breaking, a recent study on the buds of Japanese pear (*Pyrus pyrifolia* Nakai) collected in the pre-bud breaking period phase were used to identify the proteins with the result that the majority of the identified proteins (more than 20 proteins) were involved primarily in the oxidation-reduction process [5]. Among these, catalase, l-ascorbate peroxidase and peroxidase were identified, enzymes known to be very closely-involved in oxidoreductase activities [5]. The decomposition of H_2_O_2_ by catalase and peroxidase seems to be relevant in the context of the redox reactions involved in the transition of the endodormancy phases [5]. The dormancy related data on protein expression of the *Pinus sylvestris* L. *Var. Mongolica litv.* apical buds also reveal that ascorbate peroxidase may be involved in the initiation of bud dormancy, whereas caffeoyl-CoA *O*-methyltransferase was one of the biomarkers suggested to be involved in the initiation of bud dormancy release [11].

On the other hand, an evaluation based on a corresponding metabolic approach has not yet been fully addressed [12]. Sugars, especially sucrose, oligosaccharides, amino acids, phenolic compounds and organic acids were among the relevant metabolites connected with bud opening in conifer buds under forced deacclimation (artificially induced spring) [12]. Mass-spectrometry-based metabolome analyses are becoming more relevant for the profiling of known and unknown plant metabolites (targeted/untargeted) [13,14]. Accordingly, studies related to cold acclimation in *Picea sitchensis* and *Picea obovate* have been addressed using gaschromatography/massspectrometry (GC/MS)-based metabolite profiling [15,16,17], but there is still a lack of data for trees economically relevant to fruit and berry farming. A long-term study obtained data on plant metabolites during winter rest, and ontogenetic development in sweet cherry buds (*Prunus avium* L.) of cultivar “Summit” to determine which of these plant metabolites could serve as biomarkers for the different phase transitions. The focus in this study was on the different metabolites involved in oxidation-reduction processes during bud dormancy. Buds collected from the cherry trees of the cultivar “Summit” were therefore analyzed by mass-spectrometry-based methods. We report here on data derived from the seasons 2014/15–2016/17.

## 2. Results and Discussion

The different phases of para-, endo- and eco-dormancy, and ontogenetic development for cherry flower buds of the cultivar “Summit” were characterized as recently reported [1,2]. The picking period (PR–LF, PR: picking ripeness, LF: Leaf Fall) is characterized by the flower buds’ inability bloom. On average, this phenophase lasts for about 4 months, where the period S1–LF (S1: first sampling date) represents an integral part of it lasting 6 weeks (season 2014/15–2016/17) [6]. A shorter period of endodormancy (LF–t_1_) follows, ending with its release at the timing t_1_, which can be determined by observing the harvested twigs under controlled conditions in a climate chamber experiment [1,2]. The period of endodormancy lasts for 21–28 days as observed for the three seasons 2014/15 to 2016/17 [6]. Subsequently, a relatively long period of ecodormancy (t_1_–t_1_*; 63–98 days, [6]) can be observed up to the beginning of the ontogenetic development (t_1_*). This date can be related to a steady and continuous increase of the water content of the buds, related to rising air temperatures [2]. In the following period, the ontogenetic development-related “bud swelling” (SB), “side green” (SG), “green tip” (GT), “tight cluster” (TC) and “open cluster” (OC) dates were documented [6]. The duration (d) and the average temperatures for the different stages are given in [6] (see also Supplementary Tables S1 and S2).

In the present work, focus has been placed on those metabolites involved in oxidation-reduction processes during aforementioned phases and their change in the course of dormancy and ontogenetic development for cherry flower buds of the cultivar “Summit”.

### 2.1. Characterization of the Phenolic Compounds and the Antioxidative Potential of the Flower Buds

**Figure 1:**
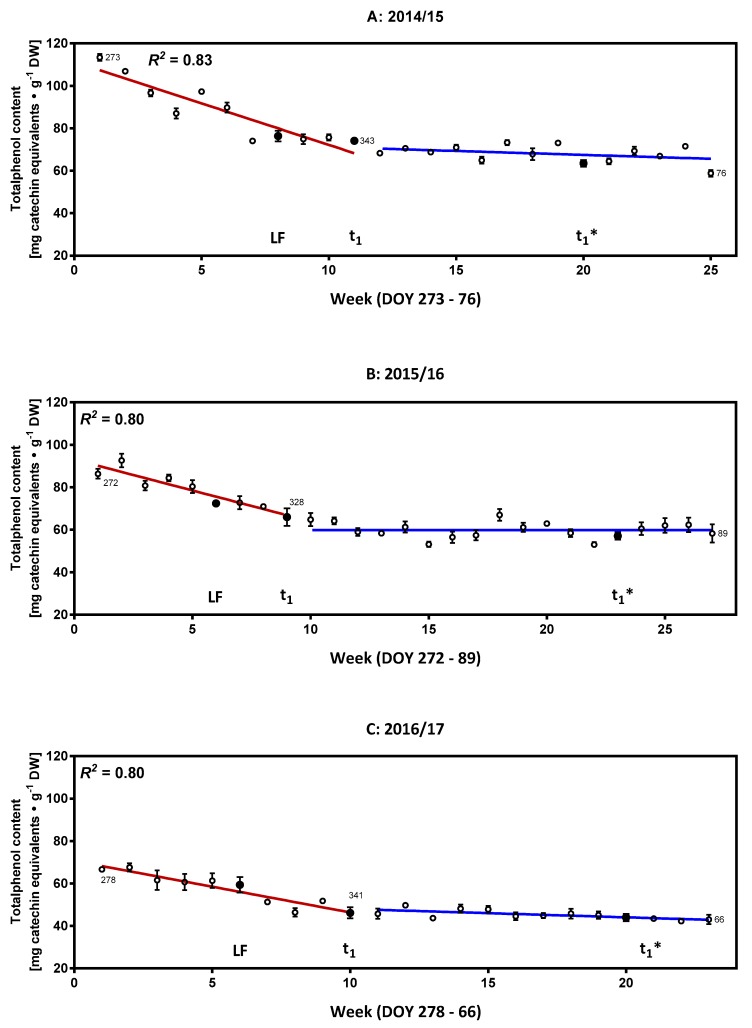
Weekly and developmentally orientated changes of total content of phenolic compounds. (**A**) Season 2014/15; (**B**) Season 2015/16 and (**C**) Season 2016/17. Solid black circles indicate key developmental milestones. Abbreviations: LF, leaf fall; t_1_, end of endodormancy; t_1_*, beginning of ontogenetic development; DOY, day of year; DW, dry weight.

**Figure 2:**
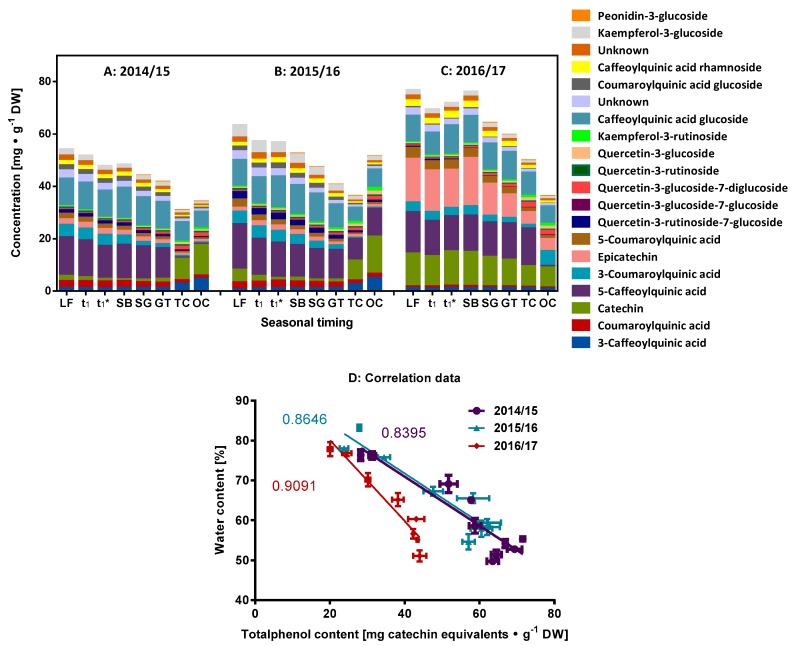
Composition of the main phenolic compounds at different developmental milestones, as determined by HPLC. (**A**) Season 2014/15; (**B**) Season 2015/16 and (**C**) Season 2016/17. (**D**) Correlation of the content of total phenolic compounds with the water content of the buds for the phenophase t_1_*–OC. Abbreviations: LF, leaf fall; t_1_ = endodormancy release, t_1_* = beginning of ontogenetic development, SB = swollen bud, SG = side green, GT = green tip, TC = tight cluster, OC = open cluster; DW, dry weight.

Based on data available from previous studies [5,11,12,17], we first focused on the group of phenolic compounds, which are known to partake in many different redox reactions. A high degree of compartmentation of phenylpropanoid and flavonoid compounds is generally observed and they may accumulate in vacuoles or are covalently integrated into plant cell membrane-like tissues [18]. The determination of the total phenolic compounds with 60% aqueous methanol using Folin-Ciocalteu phenol reagent is shown for the seasons 2014/15 to 2016/17 in Figure 1 for the three periods S1–t_1_* with the highest values at the beginning of the data sampling (113.4 ± 1.6, 92.6 ± 3.2, 67.6 ± 1.9 mg catechin equivalents∙g^−1^ DM for the seasons 2014/15, 2015/16 and 2016/17 respectively). This high content (7–11% DM) makes the phenolic compounds one of the major group of redox metabolites present in the cherry flower buds of the cultivar “Summit”. At the beginning of the dormancy, there is a decline in the content of phenolic compounds with a strong correlation (*R*^2^ = 0.80–0.83) until the developmental milestone t_1_, indicating that these compounds are either transported towards other tissues, metabolized or degraded. In the following long period of ecodormancy (77 ± 18.5 d) [6], relatively constant values for the content of the total phenolics were observed (mean values were 68.0 ± 3.2 for 2014/15; 59.8 ± 3.8 for 2015/16; 45.3 ± 2.2 for 2016/17 in mg catechin equivalents∙g^−1^ DM, see also Figure 1). Figure 2 shows the allocation and composition of the specific phenolic compounds in cherry buds. The main groups of phenolic compounds determined by the high performance liquid chromatography (HPLC) were caffeoylquinic acids (chlorogenic acid isomers/derivatives), coumaroylquinic acid (isomers/derivatives), catechins, quercetin and kaempferol derivatives, and one peonidin derivative (Figure 2A–C). Comparing this data for the two methods presented here (HPLC and total phenol content) also indicates that a few relevant phenolic compounds might be present at low concentrations, as discussed later in the results of the untargeted analysis (see also Supplementary Figure S1). In buds of black currants, hydroxycinnamic acids constituted the major group of phenolic acids and revealed two major phenolic acids, both being chlorogenic acid isomers (3-*O*-caffeoylquinic acid and 5-*O*-caffeoylquinic acid), just as is observed in the present study [19]. The data are also concordant with regard to the prevalent flavonoids (catechin/epicatechin, quercetin, kampferol derivatives) found in the present study. HPLC separation with tandem mass spectrometry (MS/MS) identification and subsequent evaluation of concentrations of individual phenolics in free, conjugated, and bound forms in the 7 sweet cherry cultivars (fruits) additionally revealed that the major compounds were chlorogenic acid isomers/derivatives, catechin and epicatechin [20]. Generally, the phenolic compounds detected in the present study were also found in cherry fruits of different cultivars, although there were differences in their relative levels [20,21]. During the seasons 2014/15 and 2015/16, the chlorogenic acid isomers/derivatives represent the major metabolites. In contrast, for the season 2016/17 higher amounts of catechin and epicatechin were observed. The factors responsible for the altered composition in season 2016/17 have not yet been determined and will require further long-term investigations. Changes in the composition are also reported to be induced by other environmental factors such as light or nutrient supply [22]. Further investigations about the changes in phenolic compounds, e.g., conversation reactions and glycosylation pattern in respect of dormancy/ontogenetic development are necessary. Generally, the distribution of the phenolic compounds changes only slightly up until the developmental milestone GT, following which higher differentiation in the composition was observed. Specifically, for the seasons 2014/15 and 2015/16, the concentration of flavonoid glycosides declined towards the milestone TC, then either increased or declined further obviously as an interaction with the environment (e.g., temperature, see also Supplementary Table S2). While the content of most compounds decreased over time, suggesting a dilution effect due to growth, the corresponding concentrations of kaempferol-3-rutinoside and quercetin-3-glucoside-7-diglucoside increased. Both are relatively complex compounds revealing the loss of antioxidant activity during development [23]. Furthermore, a high shift in some compounds was seen particularly in the last stages TC and OC, where catechin and neochlorogenic acid contents were enhanced while chlorogenic acid, known for its high antioxidant activity, was reduced [24]. Peonidin-3-glycoside was present in all developmental stages from SG to OC, representing the pinkish color of cherry flowers.

The acquired data does not allow for the determination of a consistent individual marker for the individual milestones when considering all three investigated seasons for the different stages of sweet cherry bud dormancy and ontogenetic development. The total phenol content generally decreases significantly after reaching the milestone SB (Supplementary Figure S2). In the same context, it was shown that the beginning of ontogenetic development was related to a steadily rising water contents in the buds, induced by steadily increasing air temperatures above the freezing point. Here, water content in the bud could be presented as a simple but very effective marker to define t_1_* [2]. Therefore, the content of total phenolic compounds was related to the water content of the buds for the phenophase t_1_*–OC, resulting in high negative correlation values (*R*^2^ = 0.84–0.91, Figure 2D). Rising water content seems to facilitate the dilution of the phenolic compounds.

In summary, the results from this section document the de-accumulation of phenolic compounds (transport, metabolism, degradation) until the end of dormancy (t_1_), after which a long period of constancy can be observed until t_1_* followed by a final phase of further decrease to the phenophase OC, correlating with increasing water content. Catalase, l-ascorbate peroxidase and peroxidase were identified to be closely-involved in oxidoreductase activities [5]. The decomposition of H_2_O_2_ by catalase and peroxidase seems to be relevant in the context of the redox reactions involved in the transition of the endodormancy phases [5]. The decomposition of hydrogen peroxide to water and oxygen may protect the cell from oxidative damage by reactive oxygen species. On the other hand, the phenolic compounds may interact with their strong antioxidative properties in a similar way, partly explaining their decrease in the first phase (S1–LF). Later, after reaching t_1_*, the increasing water content may increase the concentration of solved phenolic compounds and facilitate their dilution.

**Figure 3:**
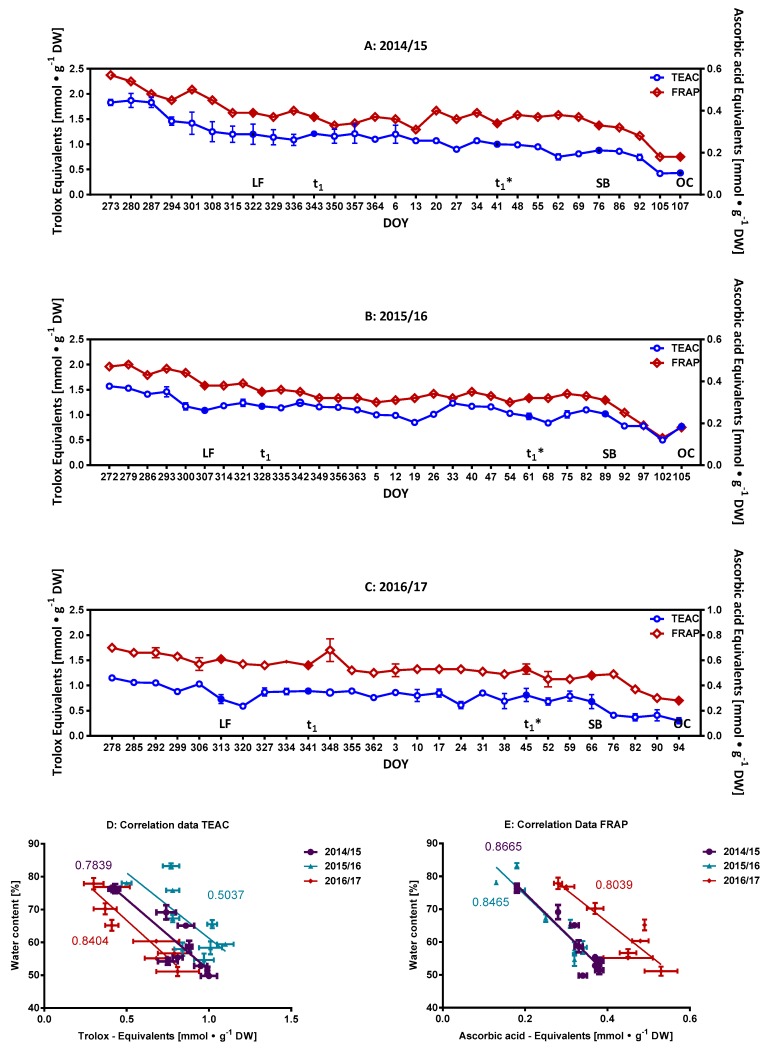
Weekly and developmentally orientated changes in anti-oxidative potential. Data is presented as Trolox (TEAC) or ascorbic acid (FRAP) equivalents. (**A**) Season 2014/15; (**B**) Season 2015/16 and (**C**) Season 2016/17. Filled symbols indicate key developmental milestones; (**D**) Correlation of water content v/s TEAC values; (**E**) Correlation of water content v/s FRAP values; both for the buds of the phenophase t_1_*–OC. Abbreviations: LF, leaf fall; t_1_ = endodormancy release, t_1_* = beginning of ontogenetic development, SB = swollen bud, SG = side green, GT = green tip, TC = tight cluster, OC = open cluster, DOY, day of year; DW, dry weight.

To follow-up this line of discussion, antioxidative activity was monitored for the whole investigative period. Figure 3A–C documents the corresponding results for the three seasons of 2014/15–2016/17. The antioxidant capacity of the same bud extracts as used for the above-mentioned analysis of the phenolics were evaluated by the TEAC and FRAP assays, both methods functioning on the basis of electron transfer during redox reactions. A positive correlation was previously found between antioxidant activity and total free phenolics of sweet cherry fruits [20]. A similar trend can also be observed for the buds in this study. The anti-oxidant capacity decreases with the content of phenolic compounds until t_1_, remains more or less stable over the period of ecodormancy (t_1_–t_1_*), then decreases further during the ontogenetic development of the sweet cherry buds (t_1_*–OC). The collected data allows one to differentiate these three phases (S1–t_1_; t_1_–t_1_*; t_1_*–OC) with common regions of transition periods. The corresponding correlation factors for the observed trends are provided (Supplementary Figure S3). The data also shows a better correlation between the content of total phenolic compounds and antioxidative activity when the latter were recorded with the ascorbic acid (FRAP) equivalents (*R*^2^ = 0.86–0.98), as compared to those values determined with Trolox (TEAC) (*R*^2^ = 0.70–0.81). Further, a negative correlation can be seen from t_1_* to OC for the between TEAC and FRAP values and the rising water content of the buds (Figure 3D,E). This result further underlines that the major group of phenolic compounds additionally imprints itself on the observed changes in the antioxidative activity, especially with regard to the dilution caused in the latter stages of the ontogenetic development.

### 2.2. Monitoring of Ascorbate/Dehydroascorbate during Dormancy

**Figure 4:**
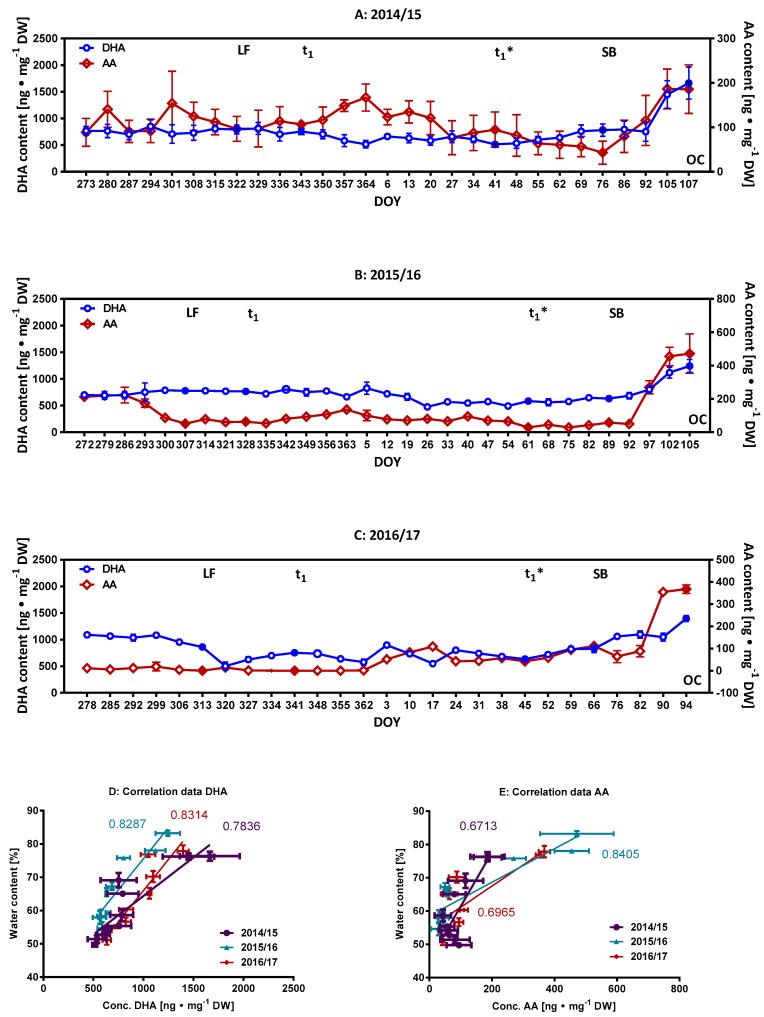
Weekly and development orientated changes of the content of AA and DHA content. (**A**) Season 2014/15; (**B**) Season 2015/16 and (**C**) Season 2016/17. Filled symbols indicate developmental milestones; (**D**) Correlation of water content v/s DHA values; (**E**) Correlation of water content v/s AA values; both for the buds of the phenophase t_1_*–OC. Abbreviations: AA, ascorbic acid; DHA, dehydroascorbic acid; LF, leaf fall; t_1_ = endodormancy release, t_1_* = beginning of ontogenetic development, SB = swollen bud, SG = side green, GT = green tip, TC = tight cluster, OC = open cluster, DOY, day of year; DW, dry weight.

Previous data has demonstrated increasing H_2_O_2_ content in floral buds of pear cultivars (*Pyrus pyrifolia*) during the pre-breaking period of endodormancy, and the possible involvement of ascorbate peroxidase as a catalyst to the reduction of H_2_O_2_ with ascorbate as an electron donor. Thus, ascorbic acid (AA) and its oxidation product dehydroascorbic acid (DHA) were identified as potential components of redox reactions [5,25]. Both are believed to be involved in preventing free radical toxicity and belong to the protective enzyme system of the glutathione-ascorbate cycle [5]. The amounts of AA (Figure 4A–C) in cherry flower buds were relatively low as compared to those of phenolic compounds, and varied seasonally in a time dependent manner (2014/15: 57–187; 2015/16: 28–471; 2016/17: 0–368 ng∙mg^−1^ DW). The content of the oxidized form DHA varies between 475–1662 ng∙mg^−1^ DW (Figure 4A–C). Though no other data are available from sweet cherry flower buds, it is notable that the AA content of fresh fruits was quantified at 7 ± 15 mg/100 g and also found to vary between the different growing seasons [21]. No consistent pattern in development orientated changes can be derived for the three seasons with regard to AA and DHA content, except that their content increases from SB (thus no marker for t_1_*) in sweet cherry buds. A positive correlation was found between the rising water content and either DHA or AA in the buds for the phenophase t_1_*–OC (Figure 4D,E; *R*^2^ = 0.67–0.83). Therefore, their DHA or AA content may also serve to characterize the period t_1_*–OC, where oxidative processes may prevail as indicated by the correspondingly high content of DHA. Similarly, the revival of AA-synthesis suggests that AA becomes one of the main redox active metabolites as the content of phenolic compounds decreases.

A possible involvement of ascorbate peroxidase in catalyzing the reduction of H_2_O_2_ with ascorbate as an electron donor has been described for the initiation period of endodormancy [5,25]. For the seasons 2015/16 (DOY 272–307), an initial decrease in the content of AA up until LF (induction of endodormancy) was noted, but which could not be verified for the seasons 2014/15 or 2016/17. In comparison, no corresponding increases in DHA values were found (more or less constant values) up to LF for the seasons 2014/15–2015/16, although a decrease was noted for the season 2016/17 over the same period. Subsequent to LF, constant values for both AA and DHA (LF–t_1_) were noted. DHA is generally actively imported, with the help of glucose transporters, into the endoplasmic reticulum of cells, where it can be reduced back to ascorbate by glutathione and/or other thiols. We presume that an equilibrium is reached between the contents of AA and DHA and the corresponding actions of the protective enzyme systems of the ascorbate and glutathione cycle.

### 2.3. Role of Reducing Sugars in Dormancy

The aldehyde functional group in reducing sugars can also partake in redox reactions on basis of electron transfer. Glucose is generally broken down during cellular respiration via an electron transport chain involving oxidative phosphorylation. It has also been observed that some relevant changes occur to glycolysis during dormancy, where glucose may be converted into pyruvate, generating small amounts of adenosine triphosphate (ATP) and nicotinamide adenine dinucleotide (NADH) [5]. A total of the eight enzymes are involved in glycolysis, all of which were identified as expressed proteins during a study involving endodormancy breaking in pears [5]. An additional label-free quantitative proteomics study also indicated the central role of glycolysis where many enzyme driven processes (six enzymes: enolase, triosephosphate isomerase, fructosebisphosphate aldolase, alcohol dehydrogenase, glyceraldehyde 3-phosphate dehydrogenase, and 3-phosphoglycerate kinase) are upregulated during the dormancy stage in terminal poplar buds [26]. In the same study, 74 significantly altered proteins were identified, most of which are involved in carbohydrate metabolism (22%), redox regulation (19%), amino acid transport and metabolism (10%), and stress response (8%) [26]. On the other hand, glycosylation can significantly improve the solubility, stability, and bioactivity of phenolic compounds. Such processes may also occur in view of the high number of phenolic compounds/metabolites found in the buds of cherry blossoms [27]. Sugars and other compatible solutes exert cryoprotective properties and accumulate during frost hardening [12]. In this same context, a recent report details the role of abscisic acid and sucrose as two important metabolites that can help to identify the date of endodormancy release in sweet cherries [1]. Based on this background, the next targeted group of redox metabolites was identified as reducing sugars.

**Figure 5:**
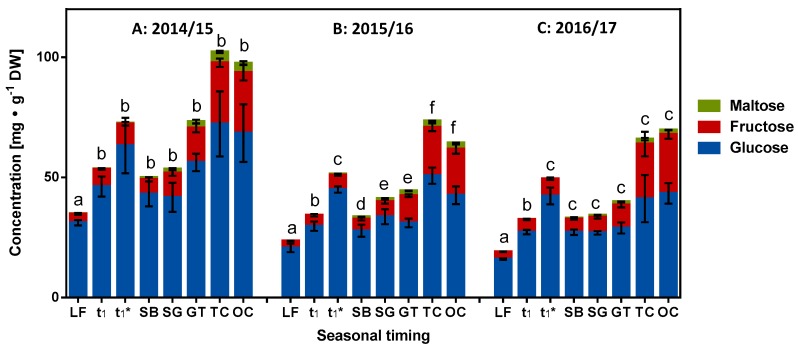
Composition of the reducing sugars at the different developmental milestones as determined by HPLC. (**A**) Season 2014/15; (**B**) Season 2015/16 and (**C**) Season 2016/17. Abbreviations: LF, leaf fall; t_1_ = endodormancy release, t_1_* = beginning of ontogenetic development, SB = swollen bud, SG = side green, GT = green tip, TC = tight cluster, OC = open cluster; DW, dry weight. Different small letters in each season (a–f) indicate significantly different mean values of the total reducing sugars from the previous or subsequent values (Tukey s multiple comparisons test, *p* ≤ 0.05, *n* = 4) for each milestone.

**Figure 6:**
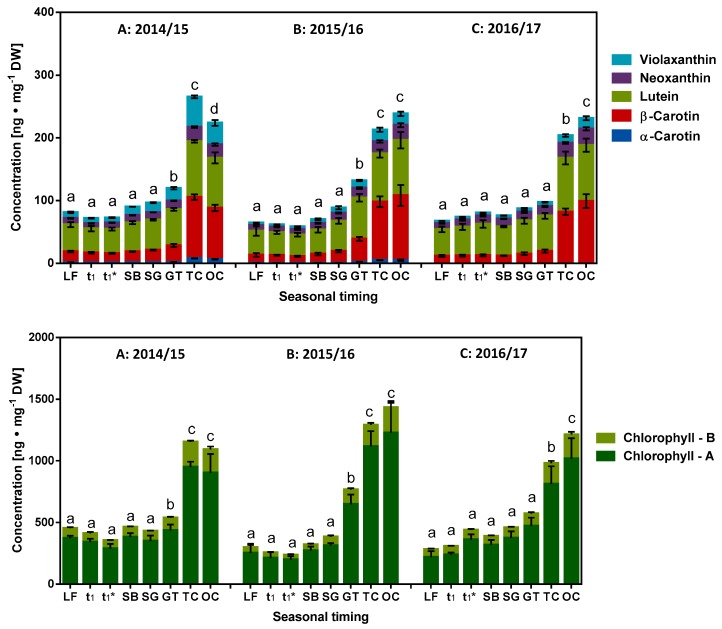
Composition of the main carotenoids and chlorophylls during the different milestones of the development stages as determined by HPLC. (**A**) Season 2014/15; (**B**) Season 2015/16 and (**C**) Season 2016/17. Abbreviations: LF, leaf fall; t_1_ = endodormancy release, t_1_* = beginning of ontogenetic development, SB = swollen bud, SG = side green, GT = green tip, TC = tight cluster, OC = open cluster; DW, dry weight. Different small letters in each season (a–d) indicate significantly different mean values of the total carotenoid/chlorophylls from the previous or subsequent values (Tukey's multiple comparisons test, *p* ≤ 0.05, *n* = 4) for each milestone.

A quantification method using HPLC equipped with an evaporative light-scattering detector was established and complemented by identification and verification via HPTLC. Information regarding the latter is given in the supplementary information (Supplementary Figure S4). The provided data show the derivatization with the aniline diphenylamine o-phosphoric acid reagent and visualization with white light illumination, with UV at 366 nm as well as the HPTLC-ESI^+^-MS spectra of the main sugars. Initially, only the reducing sugars glucose and fructose were found in the bud samples at different milestones. After the end of ecodormancy stage (t_1_*), maltose was also identified. Figure 5 shows the composition of the reducing sugars at the different developmental milestones as determined by HPLC. The detailed course of weekly and developmentally orientated changes to the content of reducing sugars is given in the supplementary Figure S5. Consistent trends for the different phenophases can be elucidated for all three seasons: the content of glucose and fructose initially decreases to reach the lowest values (20–32 mg∙g^−1^ DW) in vicinity of the LF indicating the beginning of endodormancy. Subsequently, their content increases during endodormancy and ecodormancy to reach maximum values during the phase t_1_–t_1_* (2014/15–DOY 27 = 80 mg∙g^−1^ DW; 2015/16–DOY 5 = 66 mg∙g^−1^ DW; 2016/17–DOY 24 = 55 mg∙g^−1^ DW; supplementary Figure S5). From t_1_* to SB, there is a decrease in the content of reducing sugars. Then, after the visually perceptible ontogenetic development of sweet cherry buds, SB–OC, a marked increase in the content of reducing sugars is observed. This data reveals that there is a highly regulated metabolism of glucose until LF, confirming the observations made in other studies regarding glycolysis during dormancy [5,26]. Subsequently, an accumulation of reducing sugars is initiated, eventually resulting from breakdown of oligo- and polysaccharides (LF–t_1_*). With the initiation of photosynthesis (accompanied by an increase in the content of the chlorophylls; Figure 6), their synthesis is again revived. Concluding this part, the lowest content of reducing sugars was found at LF and maltose is unfortunately no marker for t_1_*, because it starts to increase significantly after t_1_* at (2015/16) or after SB (2014/15, 2016/17) [1].

### 2.4. Role of Fat Soluble Redox Metabolites in Dormancy

The investigations until now has been limited to redox metabolites with high to medium polarity, now a few selected more hydrophobic metabolites with reported potential antioxidative properties will be considered such as carotenoids [28] and chlorophyll derivatives [29]. In this context, abscisic acid has also been discussed as one of the central regulators of bud dormancy [3] and its biosynthesis appears to be well connected to the carotenoid pathways with participation of neoxanthin and violaxanthin as precursors [30]. In this context, it was also recently shown [6] that the results of the analysis for the content of neoxanthin and violaxanthin in the seasons 2014/15 to 2016/17 were unaffected over the different phases endo- and ecodormancy and their individual contribution to ABA synthesis was not clearly discernible.

The composition of the main carotenoids and chlorophylls during the course of the development stages were determined by HPLC and only data on selected milestones are shown in Figure 6. Their content remained more or less constant during the different stages of dormancy (para-, endo- and ecodormancy) until t_1_*. Subsequently, with the beginning of visually perceptible ontogenetic development in sweet cherry buds (SB–OC), their content increases sharply similar to that of chlorophylls (Figure 6). The participation of lipophilic metabolites in the redox processes during dormancy remains largely incomplete, since our focus was limited to redox metabolites with high to medium polarity.

### 2.5. Non-Targeted Analysis of the Metabolites

The targeted analysis of selected redox active metabolites was discussed in the preceding sections. In the following, an untargeted approach was applied for selected developmental milestones for the season 2014/15 (LF, t_1_, t_1_*, SB and OC), using a data set of 20 analysis samples representing *n* = 4 for each milestone, to identify further relevant metabolites. Altogether, 5469 from an initial 13,258, and 411 from an initial 1704 entities were respectively selected from MS-profiling in negative and positive modii. The quality of the filtered entities (based on flag/frequency settings) were further confirmed by statistical one-way ANOVA (*p* < 0.05). A principle component analysis (PCA) was conducted to differentiate the different milestones for the metabolites and is presented in supplementary Figure S6. The individual data sets for the different milestones were closely clustered and could be clearly separated from one another (supplementary Figure S6). Based on their molecular mass, the metabolites were identified using the Mass Hunter Metlin PCD software to give a general overview of their up- and down- regulations, and to identify further focusing areas of the corresponding targeted analysis of suitable candidates. Identification, therefore, was tentatively executed since, while isobaric substances may have the same mass, they may still be structurally completely different. The data showed a series of different molecular species that could be allocated, for the majority of the structures, to secondary metabolism (especially to those concerning the phenolic compounds), carbohydrate, lipid (phospholipids) and protein metabolism (peptides). A few interesting compounds were selected and data on them is given in supplementary Table S3. The tentative data on glutathione (reduced and oxidized status) supports the observation made for ascorbic acid, suggesting a hand in hand involvement of these in the protective enzyme system of the ascorbate and glutathione cycle [5]. An increased glutathione oxidation at the milestones LF and t_1_ seems to occur and with the progression to ecodormancy it decreases with a corresponding elevated fold change for reduced glutathione. A series of peptides (many also containing cys) were also found among the allocated metabolites. One such example (asp met trp), given in supplementary Table S3, may support glutathione in such reactions since their participation in redox reactions is likely. The data for maltotetraose supports the observations reported for sugars and oligosaccharides as they may exert cryoprotective properties and accumulate during frost hardening metabolism in endodormancy [12]. It was only upregulated at the milestone t_1_ (as compared to LF) after which it was subsequently down regulated. A large number of metabolites were allocated to phenolic compounds (kaempferol-, quercetin-, myricetin-, luteolin-, catechin- and gallocatechin as well as hydrooxycinnamic- and hydrooxybenzoic acid derivatives/conjugates). The regulation of two of these metabolites is shown as an example in supplementary Table S3, and exemplifies the need for complementary methods to the targeted method applied in this study. In Norway spruce (*Picea abies*), *p*-Hydroxyacetophenone caused trees needle-fall, retarded apical growth, and inhibited bud-sprouting in biological tests [31]. It was upregulated only at LH and may present a good marker for this milestone; further supplementary studies are needed to confirm this observation. Finally, the lipophilic metabolites still need to be evaluated, a few interesting candidates are presented in supplementary Table S3. More sensitive methods are needed to encompass such significant metabolites of low abundancy and further work will be directed to quantify them.

## 3. Materials and Methods

### 3.1. Chemicals

Liquid chromatography-mass spectrometry (LC-MS) grade solvents were used for the LC-MS/MS analysis. All the other chemicals used were of analytical grade (VWR, Darmstadt, Germany). Details to individual reference substances are given in the corresponding method description.

### 3.2. Study Design and Sampling

The details of the growth conditions and sampling are given in Götz et al. [32]. The experiments were performed at an experimental Sweet Cherry Orchard of the Humboldt-University in Berlin-Dahlem (52°28’ Northern latitude and 13°18’ Eastern longitude).

#### Bud and Twig Sampling

Four trees in the middle of the orchard were selected to collect the buds. Sampling of 3 clusters from each tree (*n* = 4) were taken weekly at random locations over the whole tree starting 30 September 2014–10 March 2015, 29 September 2015–22 March 2016, 04 October 2016–28 February 2017 and after beginning of different bud development stages at “swollen bud” (SB), “side green” (SG), “green tip” (GT), “tight cluster” (TC) until “open cluster” (OC; BBCH 56). After cutting, the buds were kept in plastic bags on ice, frozen in liquid nitrogen and stored at −80 °C until freeze-drying. Leaf fall was also noted (LF; BBCH 97; all leaves have fallen).

The release of endodormancy (t_1_) was estimated by observing twigs under controlled conditions according to [2,7,32]. The first indication for the transition from the dormant stage to the beginning of ontogenetic development (t_1_*) was determined by evaluating the bud’s water content [2,32].

### 3.3. Phenolic Compounds

#### 3.3.1. Extraction of Phenolic Compounds

Freeze dried samples (25 mg) of each sampling date were extracted with 0.75 mL 60% methanol using an ultrasonification treatment (Sonorex RR 100, Bandelin electronic GmbH & Co. KG, Berlin, Germany) for 3 min followed by incubation at 4 °C overnight (AEG Santo 60240 DT 28, Electrolux Hausgeräte GmbH, Nürnberg, Germany). The extracts were centrifuged at 9300× *g* for 10 min at 4 °C, the extraction repeated, and the supernatants pooled together and stored at −20 °C until needed.

#### 3.3.2. Total Content of Phenolic Compounds

The total phenolic content was estimated using Folin Ciocalteau procedure [24,33]. The data is expressed as mg catechin equivalents∙g^−1^ DW using an external calibration with catechin (Sigma-Aldrich Chemie GmbH, Taufkirchen, Germany).

#### 3.3.3. Determination of Antioxidative Capacity

Trolox equivalent antioxidant capacity assay (TEAC) [24,34] was applied with a few modifications, using a microplate reader (Fluostar Optima, BMG LABTECH, Ortenberg, Germany). Results were expressed as mM Trolox (6-Hydroxy-2,5,7,8-tetra-methylchroman-2-carboxylic acid, Sigma Aldrich, Steinheim, Germany) equivalents (TE). The antioxidant activity was also determined according to the Ferric Reducing Ability of Plasma (FRAP) assay as previously described [35,36]. Ascorbic acid served as reference defining a calibration curve (50–1000 µM) to obtain the corresponding ascorbic acid equivalents.

#### 3.3.4. Identification and Quantification of the Major Phenolic Compounds HPLC-DAD-ESI-MS^n^

An 1100 HPLC series from Agilent (Waldbronn, Germany) was used to determine the composition of the phenolic compounds (hydroxycinnamic acid derivatives and flavonoid glycosides) according to [37]. The separation was conducted on a Phenomenex Prodigy column (125 × 3.0 mm, ODS 3.5 µm, 100 Å, Phenomenex Ltd. Deutschland, Aschaffenburg, Germany) with a security guard C18 column (4 × 3.0 mm, ODS 3.5 µm, 100 Å) at a column temperature of 30 °C using a water/solvent gradient. Solvent A was water with 0.5% acetic acid; solvent B was 100% acetonitrile. The following gradient was used for Eluent B: 5–7% (0–12 min), 7–9% (12–15 min), 9–12% (15–45 min), 12–15% (45–100 min), 15–75% (100–105 min), 75% isocratic (105–115 min), 75–5% (115–120 min), 5% isocratic (120–123 min). The flow rate was set at 0.4 mL∙min^─1^, and the detection was monitored at 330, 360 and 520 nm. The phenolic compounds were identified as deprotonated molecular ions with characteristic mass fragment ions by HPLC-DAD-ESI-MS^n^ using an Agilent series 1100 ion trap mass spectrometer (Agilent Technologies Deutschland, Waldbronn, Germany) in negative ionization mode. Nitrogen was used as the dry gas (10 L∙min^─1^, 325 °C) in addition to nebulizer gas (40 psi) with a capillary voltage of −3500 V. Helium was used as the collision gas in the ion trap. The mass optimization for the ion optics of the mass spectrometer was performed for quercetin with *m*/*z* 301. The MS^n^ experiments were performed in auto up to MS^3^ in a scan from *m*/*z* 200–2000. The standards, 5-caffeoylquinic acid (chlorogenic acid), quercertin-3-glucoside, kaempferol-3-glucoside, isorhamnetin-3-glucoside and cyanidin-3-glucoside (Roth, Karlsruhe, Germany) were used for external calibration at their maximum wavelengths.

### 3.4. Quantification of Ascorbic Acid (AA) and Dehydroascorbic Acid (DHA) by LC-MS/MS Analysis

For extraction 25 mg of the freeze-dried bud material were mixed (Multi RS-60, Programmable rotator, Biosan, Riga, Latvia) with 1 mL of an extraction solution containing *m*-phosphoric acid (1.5%, Merck, Darmstadt, Germany) and Ethylenediaminetetraacetic acid (0.05% EDTA, Merck) for 5 min at 4 °C. The extracts were centrifuged at 7000× *g* for 10 min at 4 °C and the supernatants filtered (0.2 µm) before the analysis. An UltiMate 3000 LC system (Thermo Fisher scientific, Dreieich, Germany) coupled to a 4000 QTRAP (Sciex, Darmstadt, Germany) was used. The column Synergi 4u Hydro-RP 80A (2.0 × 150 mm, 00F-4375-B0) and pre-column (AQ C18, 4 × 2mm, AJ0-7510-S) utilized for the separations were both from Phenomenex (Phenomenex Ltd. Deutschland, Aschaffenburg, Germany). The column temperature was set at 20 °C and the injection volume at 10 µL. The mobile phases were ultrapure water (eluent A) and methanol (eluent B) both containing 0.02% formic acid and 10 mM Ammonium formiate. A flow rate of 0.25 mL·min^−1^ with following gradient was applied: 100% eluent A, 0–2 min; 100–70% eluent A, 2–5 min; 70% eluent A, 5–5.5 min; 70–100% eluent A, 5.5–5.6 min; and finally, re-equilibration of the column was from, 5.6–13 min with 100% eluent A. The ion source parameters for MS were set as follows: Polarity—Negative (ESI-); Curtain gas—30 psi; Collision gas—medium; Ion spray voltage—minus 4200V, Temperature—350 °C; Ion Source Gas ½—50 psi. Ascorbic and dehydroascorbic acid (Sigma) were used as external calibration standards (0.1–50 µg·mL^−1^) with the mass transitions *m*/*z*: 174.9/115.0 for AA and *m*/*z*: 172.9/113.0 for DHA.

### 3.5. Targeted Analysis of Reducing Sugars

The quantification of the sugars was performed by analyzing the samples with a Shimadzu HPLC system (Shimadzu Europa GmbH, Duisburg, Germany) equipped with an evaporative light-scattering detection (Shimadzu ELSD-LT II at 40 °C, Gain = 3). The extraction and quantification details are given in Chmielewski et al. (2017) [1]. The sugar identification was complemented via High Performance Thin-Layer Chromatography (HPTLC) [38] and details are given in the supplementary information S1.

### 3.6. Targeted Analysis of the Fat Soluble Redox Metabolites (Carotenoids and Chlorophylls) by UHPLC-DAD-TOF-MS

An Agilent 1290 Infinity UHPLC coupled to an Agilent 6230 Time of Flight (TOF) MS equipped with an APCI ion source in positive ionization mode was applied as previously described [14]. Shortly, 5 mg of each freeze-dried sample (*n* = 4 for each week) were extracted three times using 0.5 mL of Methanol/tetrahyhdrofuran solution (1:1, *v*/*v*, 3 ×). The extracts were shaken at 1000 rpm for 5 min at room temperature and centrifuged at 4000×*g* for 5 min at 20 °C. The extracts were evaporated in a stream of nitrogen (evaporator VLM GmbH, Heideblüchenweg, Bielefeld, Germany) and then dissolved in 0.02 mL of dichloromethane and 0.08 mL of isopropanol. Prior to further analysis, the solutions were filtered through a 0.2 μm PTFE membrane and kept at 4 °C in the autosampler. The separation was done on a YMC C30 column (100 × 2.1 mm, 3 µm, YMC Co. Ltd., Kyoto, Japan). Methanol, methyl tert-butyl ether, and water mixtures (solvent A, 81:15:4; and solvent B, 6:90:4), were applied as mobile phases with a flow rate of 0.2 mL·min^−1^. To improve the ionization, 20 mM ammonium acetate was included in the mobile phases. Following gradient was used: 100% A (10 min isocratic), 100% A to 80% A in 7 min (28 min isocratic), 80% A to 0% A in 10 min (5 min isocratic). The gas temperature (325 °C) had a flow rate of 8 L·min^−1^, the vaporizer temperature was 350 °C, and the nebulizer pressure was 35 psi. The voltage was set to 3500 V. The fragmentor voltage (175 V) was applied at a corona current of 6.5 μA. Identification was achieved by co-chromatography with reference substances and on the basis of the mass-to-charge ratio of the pseudo molecular ions: α-carotene, and β-carotene, [M + H]^+^ 537.446; chlorophyll A [M + H]^+^ 893.543; chlorophyll B [M + H]^+^ 907.522; lutein, [M − H_2_O + H]^+^ 551.425; zeaxanthin, [M + H]^+^ 569.435; and neoxanthin, [M − H_2_O + H]^+^ 583.415 and violaxanthin [M + H]^+^ 601.425 [14]. External standard calibration curves with authentic standards were used for quantification by dose−response. β-Carotene, chlorophylls A and B were from Sigma-Aldrich ChemieGmbH (Taufkirchen, Germany). α-Carotene, lutein, neoxanthin, zeaxanthin, and violaxanthin were from CaroteNature GmbH ( Lupsingen, Switzerland).

### 3.7. Non-Targeted Analysis of the Metabolites by UHPLC-Q-TOF-MS

The metabolites were monitored for the following selected specific development stages (*n* = 4 for each development stage) for the season 2014/15: Leaf fall (LF), release of endodormancy (t_1_), beginning of ontogenetic development (t_1_*), “swollen bud” (SB) and “open cluster” (OC). The method was adapted from Errard et al. (2015) [14]. Freeze dried bud material (10 mg) were extracted with 1.5 mL of a solvent mixture (aqueous methanol with formic acid—70/30/0.1%; *v*/*v*/*v*) for 5 min on ice with an ultrasonic treatment at full power followed by shaking for another 5 min at 4 °C (1400 rpm, Thermomixer compact, Eppendorf AG, Wesseling-Berzdorf, Germany). The extracts were centrifuges at 4500× *g* for 5 min at 4 °C. The supernatant was transferred to a 10 mL flask. The extraction was repeated 4 times and finally filled up to 10 mL. Aliquots (800 µL) were membrane filtered (0.2 μm PTFE) at 3000× *g* for 5 min at 4 °C and transferred to vials. The analysis was conducted on the 1290 Infinity UHPLC coupled with an Agilent 6530 Q-TOF LC/MS (Agilent Technologies GmbH, Waldbronn, Germany). Samples (5 μL) were injected into a C18 column (2.1 × 50 mm, 1.8 μm; Agilent Zorbax Entend-C18-Rapid resolution HT). Samples and column temperature were kept at 4 and 30 °C, respectively. The eluents (eluent A, 0.01% aqueous formic acid; eluent B, 0.01% formic acid in acetonitrile) were applied to separate 5 µL of the samples by a gradient of increasing eluent B from 2 to 5% over 3 min and from 5 to 85% over 7 min. The flow rate was 0.5 mL·min^−1^. An electrospray (ESI) source was applied, and spectra were collected in positive and negative ionization modes (acquisition rate, 1 spectra/s) over an *m*/*z* 100−1700 range (capillary voltage, 3.5 kV; source temperature, 300 °C; nebulizer gas flow, 8 L/min at 35 psi; skimmer, 65 V; fragmentor voltage, 175 V). The data obtained was converted and evaluated by Mass Profiler Professional (MPP; version 12.1, Agilent Technologies, Agilent, Santa Clara, CA, USA) using molecular feature extraction (Mass Hunter B.06.00, Agilent, Santa Clara, CA, USA) as described in Errard et al. (2015) [14]. The minimum absolute abundance was set at 3000 counts, retention window at ±0.2 min with mass tolerance of 20 ppm. The data were analyzed with MPP statistical analysis by one-way ANOVA (*p* ≤ 0.05; fold change ≥2 in negative modus; fold change was set at ≥1.5 in positive modus). Putative identification was performed with Mass Hunter Metlin PCD. The principle component analysis (PCA) was conducted to differentiate the different milestones for the metabolites.

### 3.8. Statistical Analysis

The data were analyzed (mean, standard deviation, Tukey’s multiple comparisons test, *p* ≤ 0.05, *n* = 4) using statistical software Prism 6 for Windows (Vers. 6.01; GraphPad Software, Inc., La Jolia, CA, USA).

## 4. Conclusions

The observations made during the course of dormancy and ontogenetic development for cherry flower buds of the cultivar “Summit” support the observation that potential metabolites/substrates for redox reactions could be an integral part of the signaling mechanisms in plants as also reported elsewhere [39]. Many studies have suggested that proteins and genes involved in the oxidation-reduction processes including the antioxidant defense system (e.g. glutathione peroxidase, superoxide dismutase, ascorbate peroxidase) might be involved in dormancy release [5,40,41]. The data e.g., on peach buds reveal that the majority of these proteins are involved in stress-response, detoxification, defense, carbohydrate metabolism and energy production [42]. The redox state of electron transport chain components and the reduction of antioxidants such as glutathione and ascorbate which can act to regulate gene expression at different transcriptional levels seem therefore to be the most relevant processes [39]. Therefore, these observations underline the statement that the redox status operates as a major integrator of cellular metabolism and is simultaneously regulated itself by metabolic processes [43]. According to the non-targeted LC MS/MS performed, we did not find any more sugars or organic acids, suggesting how energy metabolism as a whole (glycolysis but also respiration) could evolve during the different phases. Therefore, our future work combining the implications of our study and analysis of further redox metabolites such as sugars, organic acids and energy equivalents will shed light on the redox dynamics in cherry blossoms.

## Supplementary Materials

The following are available online, Supplementary information S1: Sugar identification by HPTLC; Table S1: Determination of the different phenological development stages from leaf fall (LF) in DOY (day of the year) for the seasons 2014/15 to 2016/17 as published in [2,6]; Table S2: Duration (D in d) and average temperature (T in °C) observed during the different development stages for the seasons 2014/15 to 2016/17 as published in [2,6]; Figure S1: Comparison of the weekly and development orientated changes of the content of phenolic compounds for season 2014/15 as determined by HPLC and total phenols by Folin-Ciocalteu phenol method (TP). Figure S2: Correlation (*p* = 0.008) of the content of total phenolic compounds for the milestones SB–OC of the three seasons 2014/15–2016/17. Figure S3: Correlation of the content of total phenolic compounds to the anti-oxidative potential (data presented as Trolox (TEAC) or ascorbic acid (FRAP) equivalents) for the three seasons 2014/15–2016/17. Figure S4: HPTLC separation/identification of sugars (season 2014/15) in cherry blossom bud extracts (1 µL/band; No. 29,33,34,36–39) besides standard mixture (S1–S5; 63–630 ng/band) derivatized with the aniline diphenylamine o-phosphoric acid reagent and documented at (A) white light illumination and (B) UV at 366 nm as well as (C) HPTLC-ESI^+^-MS spectra of the three main sugars. Figure S5: Weekly and development orientated changes of the content of reducing sugars. Figure S6: Principal component analysis (*p* ≤ 0.05) on the metabolites present during the different milestones of the development stages as determined by untargeted MS analysis for the season 2014/15; Table S3: Log fold change (up- and down regulation) of selected metabolites as compared to LF present during the different milestones of the development stages as determined by untargeted MS analysis for the season 2014/15.
