# Supplementary material for: Selected Plant Metabolites Involved in Oxidation-Reduction Processes during Bud Dormancy and Ontogenetic Development in Sweet Cherry Buds (Prunus avium L.)
Source: Molecules. 2018 May 17;23(5):1197. doi: 10.3390/molecules23051197 (PMC6099681; doi:10.3390/molecules23051197)
Supplement: Supplementary file 1 [file molecules-23-01197-s001.pdf]

Selected plant metabolites involved in oxidation-reduction processes during bud dormancy and ontogenetic development in sweet cherry buds (*Prunus avium* L.).

Susanne Baldermann[1,2], Thomas Homann[1], Susanne Neugart[2], Frank-M. Chmielewski[3], Klaus-Peter Götz[3], Kristin Gödeke[4], Gerd Huschek[4], Getrud E. Morlock[5], Harshadrai M. Rawel[1*]

[1] Institute of Nutritional Science, University of Potsdam, Arthur-Scheunert-Allee 114-116, 14558 Nuthetal, Potsdam, Germany
[2] Leibniz Institute of Vegetable and Ornamental Crops (IGZ), Theodor-Echtermeyer-Weg 1, 14979 Großbeeren, Germany
[3] Agricultural Climatology, Faculty of Life Sciences, Humboldt-University of Berlin, Albrecht-Thaer-Weg 5, 14195 Berlin, Germany
[4] IGV-Institut für Getreideverarbeitung GmbH, Arthur-Scheunert-Allee 40/41, D-14558, Nuthetal OT Bergholz-Rehbrücke, Germany
[5] Justus Liebig University Giessen, Chair of Food Sciences, Institute of Nutritional Science, Interdisciplinary Research Center (IFZ), Heinrich Buff Ring 26-32, D-35392 Giessen, Germany

* Correspondence: rawel@uni-potsdam.de; Tel.: + +49-33200-88-5525 / -5578

Supplementary information S1: Sugar identification by HPTLC

The sugars (all >98 %; vwr, Darmstadt, Germany) were dissolved in water together in a stock solution mixture (1 mg.mL^{-1} each). The standard solution mixture was prepared by pipetting 100 µL of the stock solution mixture together with 1.5 mL methanol in a sampler vial (63 ng·µL^{-1} each). The sample (1 µL) and standard mixture solutions (1-10 µL) were applied bandwise on the HPTLC plate silica gel 60 ((20 cm × 10 cm; Merck, Darmstadt, Germany). The following settings of the Automatic TLC Sampler 4 (ATS 4, CAMAG) were used for application of 19 tracks: dosage speed 150 nL·s^{-1}, band length 8 mm, track distance 9.5 mm, distance from lower edge 8 mm and side edge 15 mm. After tank saturation in the Automatic Developing Chamber (ADC 2, CAMAG) for 15 min, chromatography was automatically performed with 12 mL n-butanol – i-propanol – glacial acetic acid – aqueous 2 % boric acid solution 6:14:1:3 (V/V/V/V) up to a migration distance of 60 mm according to a previous method [1]. The chromatogram was dried 1 min before and 2 min after the development. For post-chromatographic derivatization, the chromatogram was immersed in the aniline diphenylamine o-phosphoric acid reagent (mixture of 70 mL each of 2 % aniline and 2 % diphenylamine solutions, both in acetone, and 10 mL *o*-phosphoric acid, 85 %) via the TLC Immersion Device (immersion time 0 s, speed 3.5 cm·s^{-1}), followed by heating on the TLC Plate Heater (120 °C, 5 min). Stored in the refrigerator, the reagent was stable for weeks. The chromatograms were documented at UV 366 nm and white light illumination in transmission/reflection mode using the TLC Visualizer. Densitometry via absorption measurement at 380 nm was performed with TLC Scanner 4 and winCATS software using a slit dimension of 6.0 mm x 0.1 mm and a scanning speed of 10 mm·s^{-1}. Non-derivatized marked zones were directly eluted with 100 % methanol at a flow rate of 0.1 mL·min^{-1} (HP 1100 ChemStation pump, Agilent, Waldbronn, Germany) via the TLC-MS Interface (CAMAG, 4 mm x 2 mm oval elution head installed) into the electrospray ionization (ESI) single quadrupol MS (ExpressIon CMS, Advion, Ithaca, NY, USA).

Supplementary Table S1

Determination of the different phenological development stages from leaf fall (LF) in DOY (day of the year) for the seasons 2014/15 to 2016/17 as published in [2,3]; t_1 = endodormancy release, t_1*= beginning of ontogenetic development, SB = swollen bud, SG = side green, GT = green tip, TC = tight cluster.

Stage	2014/15	2015/16	2016/17
**LF**	322	307	313
**t_1**	343	328	341
**t_1***	41	61	45
**SB**	76	89	66
**SG**	86	92	76
**GT**	92	97	82
**TC**	105	102	90
**OC**	107	105	94

Supplementary Table S2

Duration (D in d) and average temperature (T in °C) observed during the different development stages for the seasons 2014/15 to 2016/17 as published in [2,3]; t_1 = endodormancy release, t_1* = beginning of ontogenetic development, SB = swollen bud, SG = side green, GT = green tip, TC = tight cluster, M = mean, SD = standard deviation.

Stage	2014/15		2015/16		2016/17		M ± SD	
	D	T	D	T	D	T	D	T
LF-t_1	21	2.6	21	9.4	28	3.0	23.3 ± 4.0	5.0 ± 3.8
t_1-t_1*	63	2.8	98	3.7	70	0.6	77.0 ± 18.5	2.4 ± 1.6
t_1*-SB	35	4.1	28	4.7	21	5.8	28.0 ± 7.0	4.9 ± 0.9
SB-SG	10	6.7	3	7.4	10	5.8	7.7 ± 4.0	6.6 ± 0.8
SG-GT	6	6.6	5	12.8	6	7.6	5.7 ± 0.6	9.0 ± 3.3
GT-TC	13	8.3	5	9.2	8	9.4	8.7 ± 4.0	9.0 ± 0.6
TC-OC	2	13.5	3	11.4	4	13.7	3.0 ± 1.0	12.9 ± 1.3

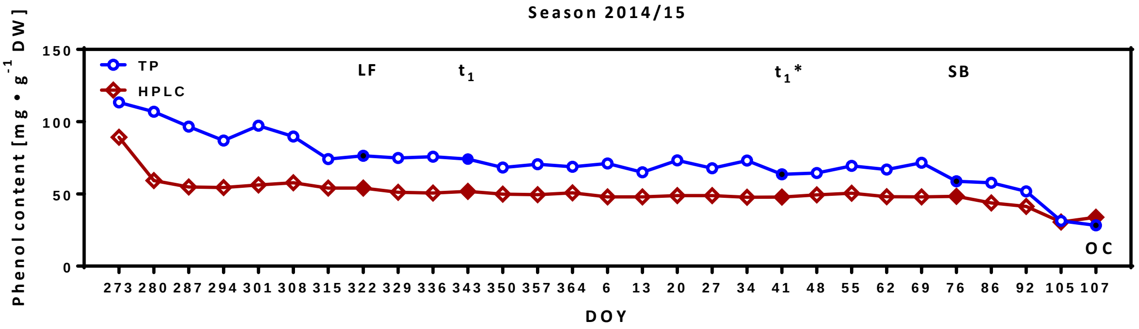

Figure S1: Comparison of the weekly and development orientated changes of the content of phenolic compounds for season 2014/15 as determined by HPLC and total phenols by Folin-Ciocalteu phenol method (TP). Filled symbols indicate the timings. Abbreviations: LF, leaf fall; t_1 = endodormancy release, t_1*= beginning of ontogenetic development, SB = swollen bud, SG = side green, GT = green tip, TC = tight cluster, OC = open cluster; DOY, day of year; DW, dry weight.

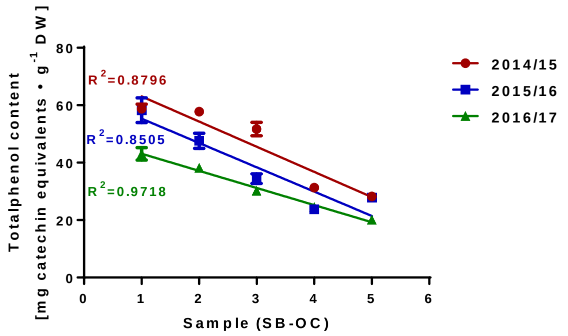

Figure S2: Correlation ($p = 0.008$) of the content of total phenolic compounds for the timings SB – OC of the three seasons 2014/15 - 2016/17. Slope is different for each data set (season). The correlation factors are for mean of 4 replications, error bars show the standard deviations. Abbreviations: SB (1) = swollen bud, SG (2) = side green, GT (3) = green tip, TC (4) = tight cluster, OC (5) = open cluster; DW, dry weight. Respective sample numbers are given in brackets.

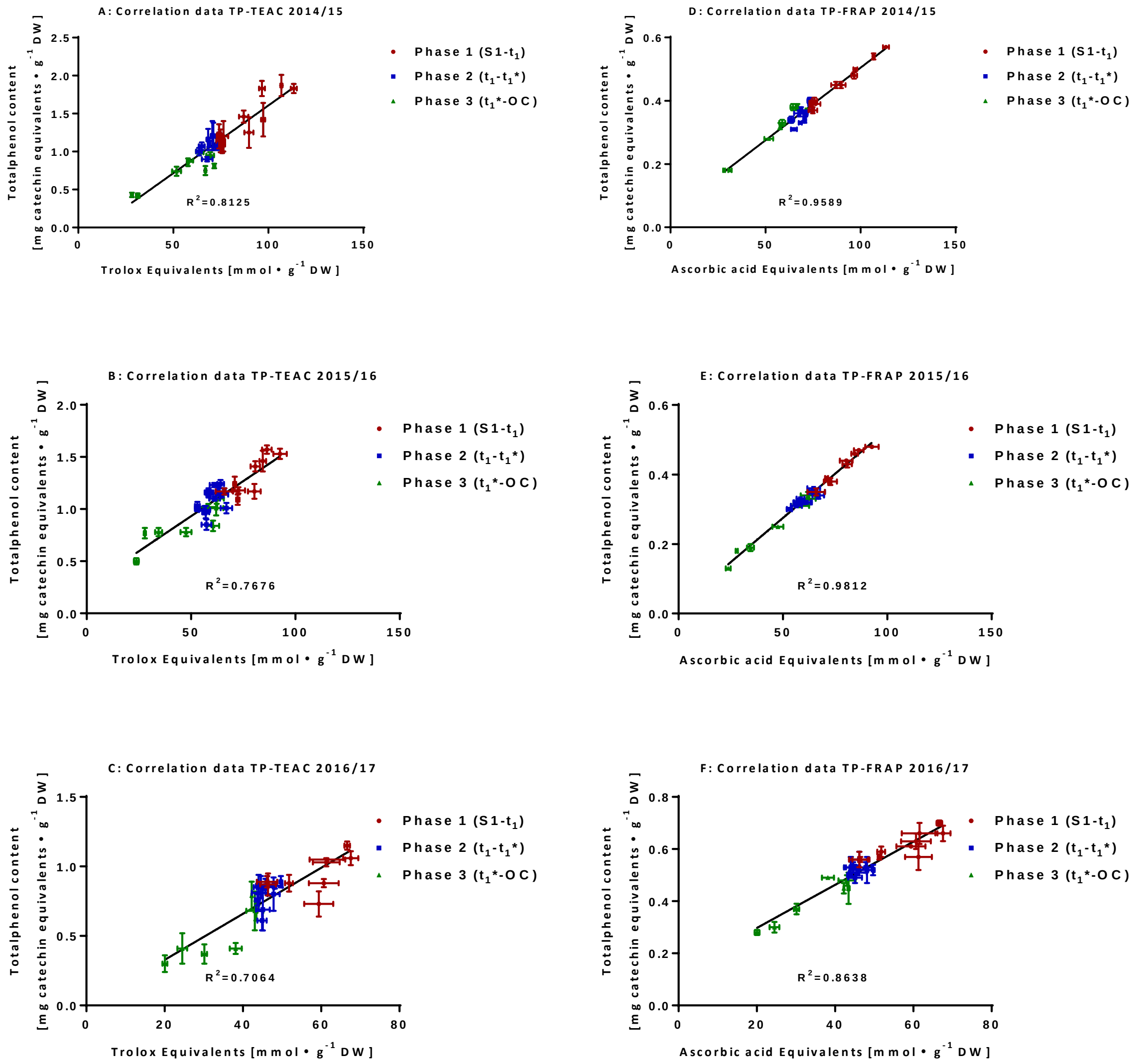

Figure S3: Correlation of the content of total phenolic compounds to the anti-oxidative potential (data presented as trolox (TEAC) or ascorbic acid (FRAP) equivalents) for the three seasons 2014/15 - 2016/17. The illustration shows the three phases of weekly and development orientated changes (S_1-t_1; t_1-t_1*; t_1*-OC). The correlation factors are for mean of 4 replications, error bars show the standard deviations. Abbreviations: S1 = first sampling date, t_1 = endodormancy release, t_1* = beginning of ontogenetic development, OC = open cluster, DW, dry weight.

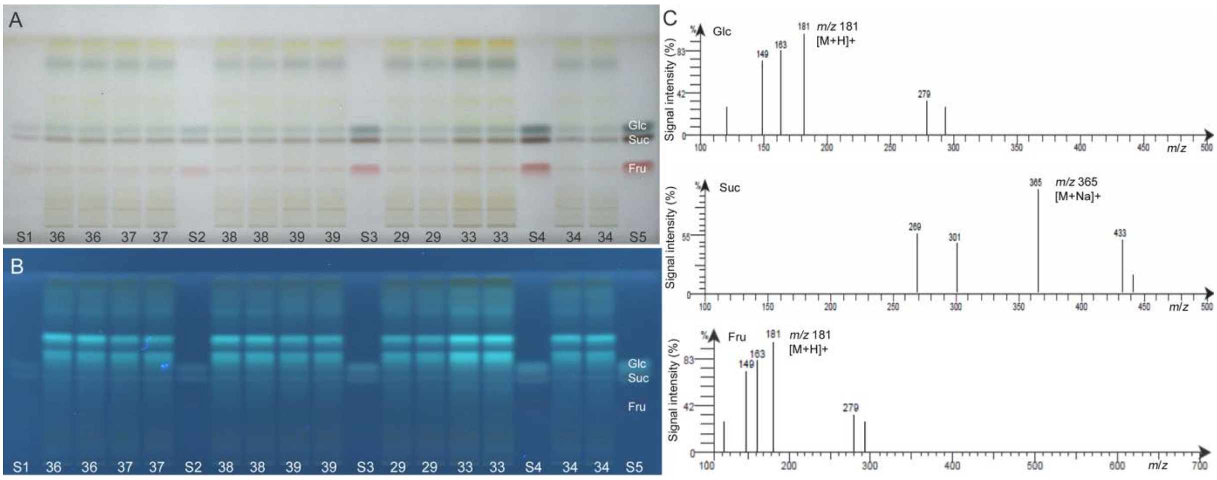

Figure S4: HPTLC separation/identification of sugars (season 2014/15) in cherry blossom bud extracts (1 µL/band; no. 29,33,34,36-39) besides standard mixture (S1-S5; 63-630 ng/band) derivatized with the aniline diphenylamine o-phosphoric acid reagent and documented at (A) white light illumination and (B) UV at 366 nm as well as (C) HPTLC-ESI^{+}-MS spectra of the three main sugars.

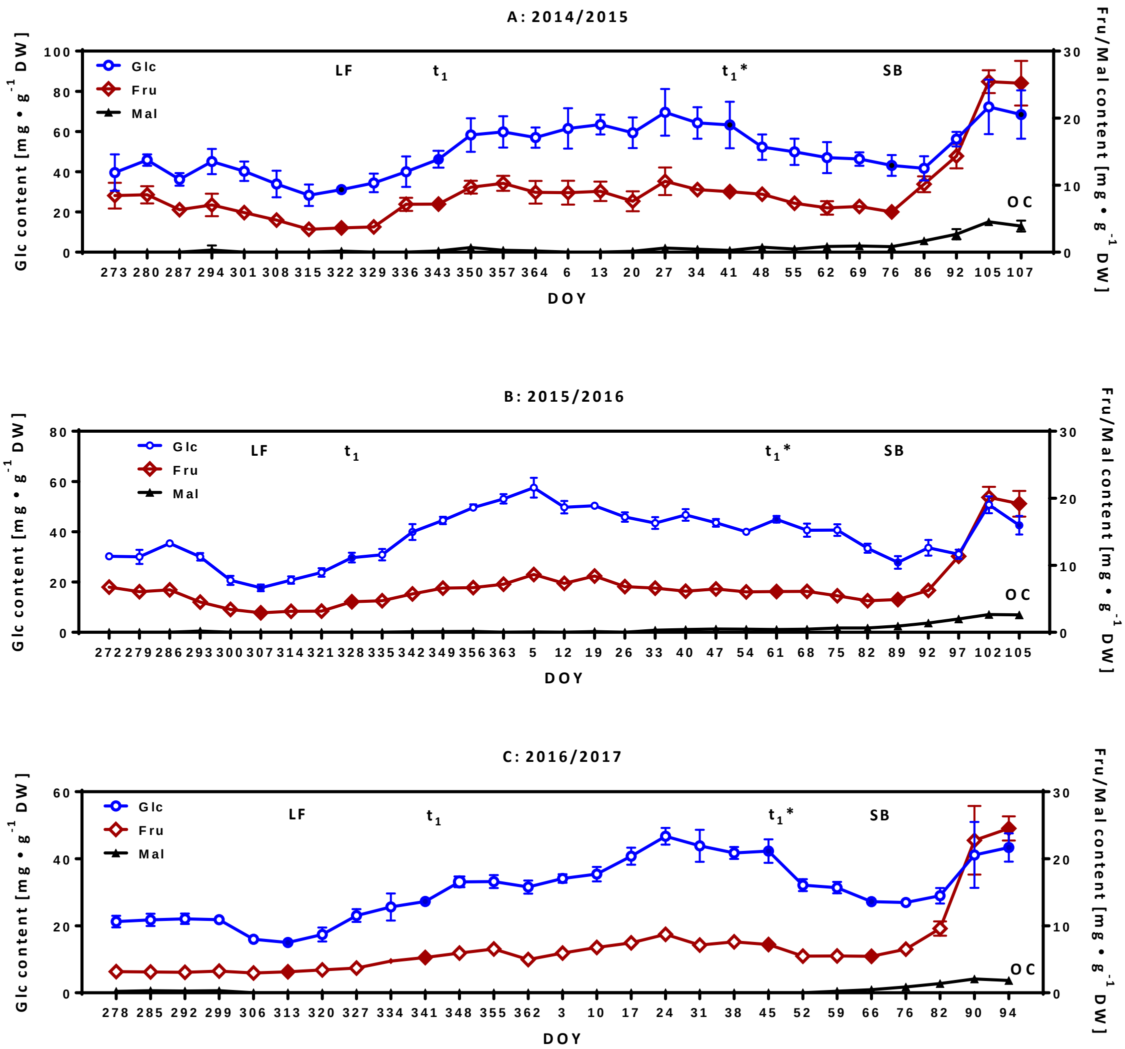

Figure S5: Weekly and development orientated changes of the content of reducing sugars. A: Season 2014/15; B: Season 2015/16 and C: Season 2016/17. Filled symbols indicate the timings. Abbreviations: Glc, glucose; Fru, fructose; Mal, maltose; LF, leaf fall; t_1 = endodormancy release, t_1*= beginning of ontogenetic development, SB = swollen bud, SG = side green, GT = green tip, TC = tight cluster, OC = open cluster, DOY, day of year; DW, dry weight.

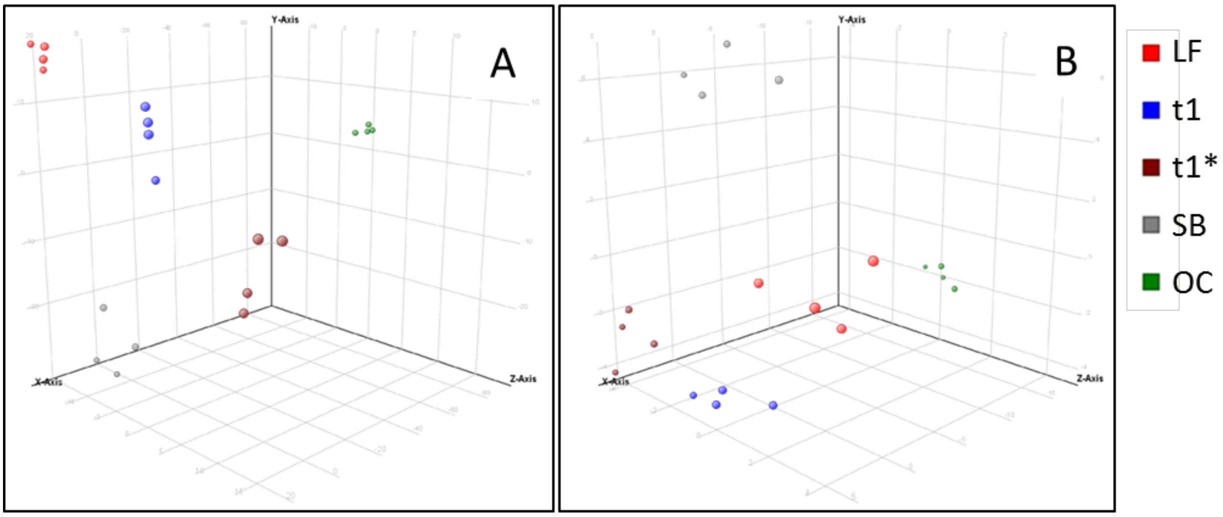

Figure S6: Principal component analysis ($p \leq 0.05$) on the metabolites present during the different timings of the development stages as determined by untargeted MS analysis for the season 2014/15; *m/z*: 100-1700. A: Fold change ≥ 2 in negative modus with PC1= 66.06% (x-axis), PC2=7.79% (y-axis) and PC3=3.72% (z-axis). B: Fold change ≥ 1.5 in positive modus with PC1= 60.8% (x-axis), PC2=9.99% (y-axis) and PC3=6.27% (z-axis). Abbreviations: LF, leaf fall; t_1 = endodormancy release, t_1*= beginning of ontogenetic development, SB = swollen bud, OC = open cluster.

Supplementary Table S3: Log fold change (up- and down regulation) of selected metabolites as compared to LF present during the different timings of the development stages as determined by untargeted MS analysis for the season 2014/15. Abbreviations: LF, leaf fall; t_1 = endodormancy release, t_1*= beginning of ontogenetic development, SB = swollen bud, OC = open cluster; n.d. = not detected.

**Compound/ Stage**	**t_1**	**t_1***	**SB**	**OC**
Glutathione, oxidzed	n.d.	-4.09	-8.27	-16.09
Glutathione, reduced	4.80	19.11	18.25	15.44
Asp Met Trp	17.58	17.98	17.97	18.66
Maltotetraose	19.41	-13.73	-18.18	-18.18
4'-Hydroxyacetophenone	-16.96	n.d.	-4.47	-16.96
Epigallocatechin	5.24	5.38	-9.22	-13.75
Methyl 3,4-dicaffeoylquinate	14.43	4.70	-4.71	-4.71
Phosphatidyl glycerol	-9.08	-9.00	-17.52	n.d.
Phytosphingosine	-4.28	8.42	-8.39	-8.39
Dehydrophytosphingosine	-9.76	-13.07	-1.58	2.40

References

1. Morlock, G.E.; Sabir, G. Comparison of two orthogonal liquid chromatographic methods for quantitation of sugars in food. *J. Liq. Chromatogr. Relat. Technol.* **2011**, *34*, 902-919.
2. Chmielewski, F.M.; Baldermann, S.; Gotz, K.P.; Homann, T.; Schumacher, F.; Huschek, G.; Rawel, H. Abscisic acid related metabolites in sweet cherry buds *J. Hort.* **2018**, *5*, 221.
3. Chmielewski, F.M.; Gotz, K.P. Identification and timing of dormant and ontogenetic phase for sweet cherries in northeast Germany for modelling purposes. *J. Hort.* **2017**, *4*, 205.
